# Compliance of Atrial Fibrillation Treatment with the Atrial Fibrillation Better Care (ABC) Pathway Improves the Clinical Outcomes in the Middle East Population: A Report from the Gulf Survey of Atrial Fibrillation Events (SAFE) Registry

**DOI:** 10.3390/jcm9051286

**Published:** 2020-04-29

**Authors:** Jakub Gumprecht, Magdalena Domek, Marco Proietti, Yan-Guang Li, Nidal Asaad, Wafa Rashed, Alawi Alsheikh-Ali, Mohammad Zubaid, Gregory Y. H. Lip

**Affiliations:** 1 Liverpool Centre for Cardiovascular Science, University of Liverpool and Liverpool Heart & Chest Hospital, Liverpool L7-8TX, UK; kubagumprecht@gmail.com (J.G.); mgk.domek@gmail.com (M.D.); marco.proietti@unimi.it (M.P.); 2 Aalborg Thrombosis Research Unit, Department of Clinical Medicine, Aalborg University, 9100 Aalborg, Denmark; 3 Department of Cardiology, Medical University of Silesia, Silesian Centre for Heart Diseases, 41-800 Zabrze, Poland; 4 Department of Internal Diseases, Diabetology and Nephrology, Medical University of Silesia, 41-800 Zabrze, Poland; 5 Department of Cardiology, Peking University Third Hospital, Beijing 100191, China; liyanguangsuper@163.com; 6 Cardiology Department, Heart Hospital, Hamad Medical Corporation, Doha 3050, Qatar; nidalasaad@gmail.com; 7 Department of Medicine, Mubarak Al-Kabeer Hospital, Jabriya, Kuwait University, Kuwait City 24923, Kuwait; dr.wrashed@gmail.com; 8 College of Medicine, Mohammed Bin Rashid University of Medicine and Health Sciences, Dubai 505055, UAE; alsheikhali@gmail.com; 9 Department of Clinical Sciences and Community Health, University of Milan, 20122 Milan, Italy

**Keywords:** atrial fibrillation, mortality, ABC pathway

## Abstract

Atrial fibrillation (AF) is associated with substantially increased risk of cardiovascular events and overall mortality. The Atrial fibrillation Better Care (**A**—Avoid stroke, **B**—Better symptom management, **C**—Cardiovascular and comorbidity risk management) pathway provides a simple and comprehensive approach for integrated AF therapy. This study’s goals were to evaluate the ABC pathway compliance and determine the main gaps in AF management in the Middle East population, and to assess the impact of ABC pathway adherence on the all-cause mortality and composite outcome in AF patients. 2021 patients (mean age 57; 52% male) from the Gulf SAFE registry were studied. We evaluated: A—appropriate implementation of OACs according to CHA_2_DS_2_-VASc score; B—symptom control according to European Heart Rhythm Association (EHRA) symptom scale; C—proper cardiovascular comorbidities management. The primary endpoints were the composite cardiovascular outcome (ischemic stroke or systemic embolism, all-cause death and cardiovascular hospitalization) and all-cause mortality. One-hundred and sixty-eight (8.3%) patients were optimally managed according to adherence with the ABC pathway. Over the one-year follow up (FU), there were 578 composite outcome events and 224 deaths. Patients managed with integrated care had significantly lower rates for the composite outcome and mortality comparing to non-ABC group (20.8% vs. 29.3%, *p* = 0.02 and 7.3% vs. 13.1%, *p* = 0.033, respectively). On multivariable analysis, ABC compliance was independently associated with reduced risk of composite outcome (HR 0.53; 95% CI 0.36–0.8, *p* = 0.002) and death (HR 0.46; 95% CI 0.25–0.86, *p* = 0.015). Integrated ABC pathway adherent care resulted in the reduced composite outcome and all-cause mortality in AF patients from Middle East, highlighting the necessity of promoting comprehensive holistic and integrated care management of AF.

## 1. Introduction

Atrial fibrillation (AF) is the most frequent cardiac arrhythmia in clinical practice and it is influenced by various underlying risk factors [1,2]. Substantial cardiovascular morbidity and mortality result from AF and AF-related complications [3]. Additionally, AF is the leading cause of disability and impaired quality of life by rising the number of hospitalizations and exacerbating other disease entities such as heart failure, stroke and dementia [4,5]. Of note, AF-related strokes are associated with more disabling or fatal complications than strokes resulting from non-AF aetiologies [6].

Over the last decades, the attitude to stroke prevention in AF has changed, with various available therapeutic options as well as validated scores and schemes for bleeding and stroke risk assessment [6,7]. As stroke prevention is the cornerstone of AF management, a comprehensive knowledge of AF risk factors and their inter-relationships are of the utmost importance. In addition, the dynamic nature of these risk factors has recently been highlighted [2].

The Atrial fibrillation Better Care (ABC) pathway for integrated care management presents a novel approach to streamline the holistic management of AF [4]. The ABC pathway provides a simple strategy for a comprehensive treatment including improvement in detection and awareness of AF, as well as dealing with AF symptoms and managing risk factors (Figure S1). Given that stroke prevention is a pivotal part of AF therapy, oral anticoagulation (OAC) is recommended for all patients, except those at low risk who are unlikely to benefit from this treatment [8,9]. ‘B’ refers to better symptom control, so effective treatment of AF symptoms concerning individualized rate and rhythm control management. ‘C’ applies to the management of comorbidities and cardiovascular risk factors [4]. Previous retrospective studies have showed the association between ABC pathway adherence and lower risk of clinical outcomes as well as decrease in health-related costs [10,11,12].

For the first time in the Middle East region, this study evaluated if AF management that is compliant or adherent with the ABC pathway was associated with improved clinical outcomes. These clinical outcomes include reducing all-cause mortality and the composite outcome of ischemic stroke or systemic embolism, all-cause death and cardiovascular hospitalization.

## 2. Materials and Methods

This was a retrospective post-hoc analysis from the Gulf Survey of Atrial Fibrillation Events (SAFE) registry dataset, which is an international, prospective, observational register of AF patients from six countries in the Gulf region of the Middle East. Detailed information on the methods has been previously published [13]. In brief, the Gulf SAFE registry was based on consecutive AF patients admitted to emergency departments from 23 participating hospitals carried out between 15 October 2009, and 30 June 2010, independently from the primary reason for admission. Patients older than 18 years qualified for inclusion if they had >30 s AF on a 12-lead resting electrocardiogram. All patients were informed about the details of the study and gave informed consent for their participation. The study was approved by the ethics committees of each institution/country.

The Gulf SAFE registry enrolled 2043 patients with AF. For the purpose of the study, we included 2021 in further analysis, omitting those with missing data about the CHA_2_DS_2_-VASc score. 

We analysed compliance with the ABC pathway components, which were defined as follows on the basis of ESC Guidelines [8] (see Supplementary Materials Figure S2 available online):

- ‘A’—‘Avoid stroke’—we identified patients at low risk of ischemic stroke (those with a CHA_2_DS_2_-VASc score of 0 in men or 1 in women) and assess whether everyone else is treated with OAC. (‘A compliant’). Patients at high risk of stroke, who did not receive OAC and those with low risk of stroke, but unnecessarily anticoagulated were considered as ‘A non-compliant’. The vast majority of patients receiving OAC in the current study were administered with vitamin K antagonists (VKA, e.g., warfarin).
- ‘B—better symptoms control’—we evaluated the occurrence of symptoms and classified them according to the European Heart Rhythm Association (EHRA) symptom scale. We assumed that patients with EHRA I or II had good control of AF symptoms (‘B complaint’) in comparison to those with EHRA III or IV, who were treated insufficiently (‘B non-complaint’).
- ‘C—Cardiovascular risk and other comorbidity optimisation’—To reduce cardiovascular risk, we evaluated appropriate treatment of the following comorbidities based on available data: hypertension (HT), coronary artery disease (CAD), peripheral artery disease (PAD) and ischemic stroke/TIA. HT assessment was based on an average of the blood pressure values at hospital admission that should be < 140/90 mmHg in order to be considered as well controlled. For other comorbidities, optimal pharmacologic management was evaluated in accordance with the current European guidelines and recommendations. (Figure S2). ‘C compliant’ means that all comorbidities were either well-controlled or treated with appropriate prevention drugs or both.

Finally, patients who met all criteria were defined as the ‘ABC group’, and those who did not meet all criteria were the ‘Non-ABC’ group.

### 2.1. Outcomes

In our analysis, we primarily assessed all-cause mortality and a composite outcome of “ischemic stroke or systemic embolism, all-cause death and cardiovascular hospitalization’. The main analysis is a comparison of the above-mentioned outcomes between AF patients with optimized, integrated care holistic management (ABC group) and those without ABC pathway adherence (non-ABC).

### 2.2. Statistical Analysis

Continuous variables were reported as mean ± standard deviation and evaluated by Student’s *t*-test or Mann–Whitney, as appropriate. Categorical variables were expressed as percentages and counts and compared using Pearson’s chi-square test or Fisher’s exact test, as necessary.

The major analyses included comparisons of the clinical outcomes between the two study groups: ABC-group and non-ABC group, reflecting the ABC pathway compliance in the management of AF patients. Secondary analyses evaluated the association between the components of ABC pathway, when partially fulfilled, and clinical outcomes occurrence and also included analyses comparing the non-ABC group with a group of partial compliance with ABC pathway (AB vs. BC vs. AC groups). 

A logistic regression model was used to analyse the association between the groups considered and the study outcomes. Odds ratios (ORs) and 95% confidence interval (CI) were used to express the association. All multivariable regression models were adjusted for AF type, renal dysfunction, dyslipidemia, use of aspirin, and major bleeding. 

All tests were 2-tailed, and *p*-value < 0.05 was considered to be statistically significant. The analyses were conducted using SPSS version 24 software package (SPSS Inc., Chicago, IL, USA).

## 3. Results

From 2043 patients in the Gulf SAFE registry, 22 (1.1%) were excluded from the analysis due to missing data regarding CHA_2_DS_2_-VASc score. The cohort for the current study was comprised of 2021 patients, whose baseline characteristics are reported in Table 1.

In general, 168 (8.3%) patients were managed adherent to the ABC pathway. In comparison to the non-ABC adherent group, patients from ABC-adherent group were older (*p* < 0.001), had higher BMI (*p* < 0.001), CHA_2_DS_2_-VASc (*p* < 0.001) and HAS-BLED scores (*p* = 0.008). Comparing to the non-ABC adherent, the ABC-adherent group had lower systolic (*p* < 0.001) and diastolic blood pressure (*p* < 0.001), as well as lower left ventricular ejection fraction (*p* = 0.007), acquired more comorbidities and were treated with more drugs (Table 1). No relevant gender dominance was observed in either group (male: 50.8% vs. 52.9%).

### 3.1. “ABC” Pathway Compliance

**Figure 1:**
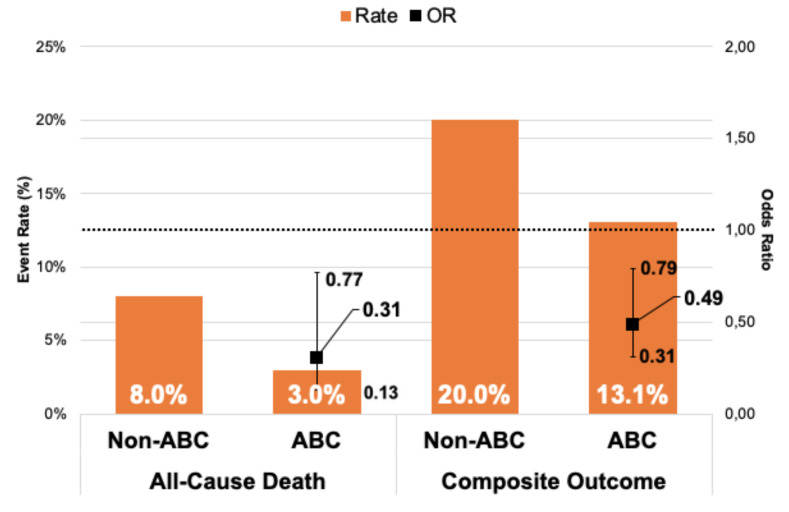
Relationship between events rates and odds ratio of clinical outcomes in 6 months follow-up. Legend: Whiskers represent 95% CI; CI = Confidence Interval; OR = Odds Ratio.

Detailed compliance of studied population to each of the ABC pathway steps is presented in Table 2. Among the subjects studied, 1118 (54.7%) subjects were treated in line with **“A”** pathway step. Classification of patients according to the ischemic stroke risk is shown in Figure 1. From the high ischemic stroke risk group, 469 (33%) patients received OAC followed by single antiplatelet therapy in 403 (28.3%), dual antiplatelet therapy 97 (6.8%), dual antithrombotic therapy 294 (20.7%) and 91 (6.4%) who were prescribed neither anticoagulation nor antiplatelet therapy. Detailed data about anticoagulation management are reported in Table S1 available online.

**“B”** criterion was fulfilled in 1518 (75.1%) patients, who were optimally managed symptomatically and hence reported no (or only mild) clinical symptoms (EHRA I-II).

AF was symptomatic significantly more often in patients with persistent/paroxysmal AF than in those with paroxysmal AF form (25.2% vs. 15%, respectively, *p* < 0.001).

**“C”**-adherence, which means that the comorbidities were either well-controlled or treated according to the international guidelines, was present in 388 (19.2%) (see Table S2).

### 3.2. Clinical Outcomes

**Figure 2:**
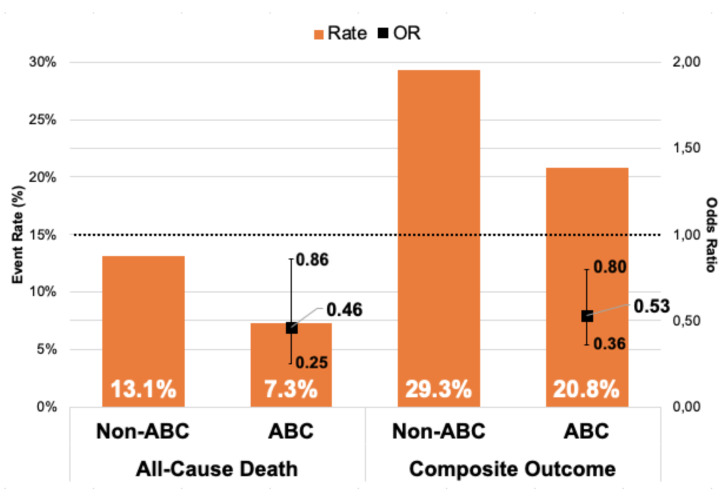
Relationship between events rates and odds ratio of clinical outcomes in 1-year follow-up. Legend: Whiskers represent 95% CI; CI = Confidence Interval; OR = Odds Ratio.

During a one-year follow-up (FU), 578 composite outcome events occurred, and 224 patients died. Patients managed adherent to the ABC pathway had lower composite outcome and all-cause mortality rates, both at 6 months and one-year FU: (i) 6 months FU (13.1% vs. 20%, *p* = 0.03 and 3% vs. 8%, *p* = 0.018, respectively); and (ii) one-year FU (20.8% vs. 29.3%, *p* = 0.02 and 7.3% vs. 13.1%, *p* = 0.033, respectively) (Figure 1 and Figure 2).

On multivariable regression analysis, ABC pathway compliance was independently associated with reduced all-cause mortality risk at both 6-months (Odds ratio (OR) 0.31; 95% Confidence Interval (CI) 0.13–0.77, *p* = 0.013) and one-year (OR 0.46; 95% CI 0.25–0.86, *p* = 0.015) FU in comparison to non-ABC-compliant group.

An adjusted analysis showed also significant association with lower risk of the composite outcome in ABC group versus non-ABC group in 6-months and one-year FU (OR 0.49; 95% CI 0.31–0.79, *p* = 0.003 and OR 0.53; 95% CI 0.36–0.8, *p* = 0.002, respectively) (Table 3).

### 3.3. Number of Fulfilled ABC Criteria and Clinical Outcomes

Apart from the B criterion for the composite outcome (OR 0.57, 95% CI0.46–0.71), none of other separate components were associated with significant difference in all-cause mortality or the composite outcome. When we analysed the relationship between partial ABC compliance and the composite outcome, AB (OR 0.75; 95% CI 0.61–0.92, *p* = 0.006) and BC (OR 0.68; 95% CI 0.50–0.92, *p* = 0.013) criteria together were associated with reduced risk (Table 4).

Neither one nor two fulfilled criteria were significantly associated with reduced risk of all-cause death in one-year FU. Only full ABC pathway compliance, i.e., all 3 criteria were fulfilled at the same time, was independently associated with a significantly reduced risk of all-cause death and the composite outcome at both 6-months (OR 0.31; 95% CI 0.13–0.77, *p* = 0.013, and OR 0.46; 95% CI 0.25–0.86, *p* = 0.015, respectively); as well as at 1 year (OR 0.49; 95% CI 0.31–0.79, *p* = 0.003 and OR 0.53; 95% CI 0.36–0.8, *p* = 0.002, respectively).

## 4. Discussion

This international cohort study from the Middle East was based on a real-world observational registry of AF patients aimed to assess the adherence of AF management according to the ABC pathway and its outcomes. The analysis demonstrated that the vast majority of patients were not managed optimally in accordance with the ABC pathway. Second, ABC integrated care was associated with a reduced risk of all-cause death and the composite outcome in comparison to non-ABC complaint group. Third, the impact of ABC pathway adherence on all-cause mortality and composite outcome reduction was independent of FU duration.

The beneficial effect coming from integrated care and holistic approach to AF treatment is documented in many randomized control trials and real-world evidence studies [14,15]. The importance of this comprehensive treatment was also underlined by the 2016 European Society of Cardiology guidelines [8]. The ABC integrated pathway meets these requirements and was proposed to simplify treatment regimens and provide practical and simple steps to guarantee holistic care for AF patients in everyday clinical practice [4]. The ABC pathway has also been incorporated into the regional primary care guidelines for AF management [12]. According to these studies, treatment adherent with the integrated care (ABC group) was associated with a significant reduction in all-cause mortality as well as the composite outcome including ischemic stroke, major bleeding, cardiovascular death, myocardial infarction and rate of total hospitalizations in comparison to non-ABC compliant group [10,11,12]. Our findings are in line with the above-mentioned studies showing reduced risks of all-cause mortality and composite outcome in the ABC group compared to non-ABC.

Moreover, the association between ABC pathway compliance and the reduced risk of the primary endpoints (all-cause death and composite outcomes) and was independent of other common risk factors and constant in 6-months or 1-year FU (Table 3). This is all the more important as AF is a disease that usually affects the elderly, who acquire several comorbidities over time [16,17,18].

In contrast to the previous studies concerning ABC pathway adherence in various populations, the current study was noticeably younger and had lower burden of comorbidities. Unlike prior studies, the ABC-adherent group in our analysis had a significantly higher ischemic stroke and bleeding risk (*p* < 0.005) [11,12,19]. Nonetheless, even in this specific population, adherence with the ABC population was strongly associated with reduced mortality and the composite outcome.

The current analysis also exposed insufficient compliance with the individual steps of the ABC integrated care in the Middle East region. Overall, the number of AF patients, fulfilling the ABC pathway criteria was eleven-times lower than the number of people who were not compliant with this management pathway (8.3% vs. 91.7%, respectively). These results obtained were more favourable than those from other research studies conducted in Asian and European populations, where the compliance with ABC pathway was fulfilled in 15.5%; 22.4% and 7.0%, respectively [11,12,19]. Although these retrospective studies varied slightly in terms of ABC pathway definition, the size of the analysed cohorts and available confounders, the overall message of integrated care is preserved, and all studies consistently reported the efficacy and superiority of ABC pathway compliance in AF patients.

### Strengths and Limitations

This is the first, multinational study evaluating the AF treatment adherence with the ABC integrated care pathway and its influence on relevant clinical outcomes in the Middle East population. The database is based on a large number of consecutive patients from various medical centres that met precisely defined criteria, which enhances the reliability of the analysis results.

Nevertheless, there are also some limitations, which should be considered. This was an observational study, some analyses might be underpowered, and some bias and deficiencies of assessment may have occurred. The BP classification was based on an average of at least 3 measurements during the hospital admission and might not reflect the actual BP targets for all individuals. Due to the retrospective nature of the study, we were not able to interfere in the treatment regimen that has changed over time. The Gulf-SAFE dataset was created in 2009 and 2010 when Non-vitamin-K Antagonist Oral Anticoagulants (NOAC) use were hardly used, therefore, the vast majority of patients receiving OAC in the Gulf SAFE registry were using VKA. Furthermore, the guidelines have changed over that time period what might have altered AF management in the Middle East and influenced the results. Nevertheless, most of the analyses were based on fundamental AF management principles which have been in textbooks for the last decade or more, that is, stroke prevention, manage symptoms with rate or rhythm control, and the optimization of comorbidities. Finally, our registry was conducted in a broad spectrum of clinical settings, in six Gulf region countries—but we would be unable to perform our analysis by individual country. Moreover, because of limited data availability, we might have missed many confounders that could impact the final results.

## 5. Conclusions

In conclusion, integrated ABC pathway adherent care resulted in the reduced composite outcome and all-cause mortality in AF patients from Middle East, highlighting the necessity of promoting comprehensive holistic and integrated care management of AF.

## Figures and Tables

**Table 1:** Baseline characteristics of patients according to adherence to ABC pathway.

Characteristics	All Patients (*n* = 2021)	ABC Group (*n* = 168)	Non-ABC Group (*n* = 1853)	*p* Value
Demographics				
Male gender, *n* (%)	1053 (52.1%)	91 (54.3%)	962 (51.9%)	0.58
Age, mean ± SD	56.74 ± 16.47	64.46 ± 11.92	56.04 ± 16.65	<0.001
Weight, mean ± SD	75.62 ± 18.29	78.98 ± 20.03	75.32 ± 18.11	<0.015
Height, mean ± SD	164.25 ± 9.40	163.14 ± 9.87	164.35 ± 9.35	0.12
BMI, mean ± SD	28.00 ± 6.38	29.63 ± 7.24	27.86 ± 6.28	0.001
Systolic BP, mmHg, mean ± SD	130.30 ± 26.46	121.6 ± 14.00	131.10 ± 27.18	<0.001
Diastolic BP mmHg, mean ± SD	79.03 ± 16.17	74.06 ± 10.25	79.50 ± 16.53	<0.001
Comorbidities, *n* (%)				
Coronary artery disease, *n* (%)	573 (28.6%)	89 (53.3%)	484 (26.3%)	<0.001
Hypertension, *n* (%)	1065 (52.7%)	117 (69.6%)	948 (51.2%)	<0.001
Dyslipidemia, *n* (%)	677 (33.8%)	103 (61.2%)	574 (31.2%)	<0.001
Heart failure, *n* (%)	557 (27.6%)	60 (35.7%)	497 (26.8%)	0.014
Ischemic stroke or TIA, *n* (%)	239 (11.8%)	19 (11.3%)	220 (11.9%)	0.83
Diabetes mellitus, *n* (%)	603 (29.8%)	86 (51.2%)	517 (27.9%)	<0.001
Chronic kidney disease *n* (%)	122 (6.0%)	6 (3.6%)	116 (6.3%)	0.016
Smoking tobacco	461 (23.0%)	32 (19.2%)	429 (23.3%)	0.219
Stroke or bleeding risk scores				
CHA_2_DS_2_-VASc, mean ± SD	2.34 ± 1.78	3.01 ± 1.53	2.28 ± 1.79	<0.001
HAS-BLED, mean ± SD	1.13 ± 1.065	1.33 ± 0.87	1.11 ± 1.08	0.008
Echocardiogram				
Left atrium diameter (mm), *n* = 1444	44.36 ± 9.11	44.61 ± 7.03	44.34 ± 9.28	0.753
LVEF, % *n* = 1490	51.23 ± 13.20	48.19 ± 14.44	51.51 ± 13.06	0.007
Medications, *n* (%) *n* = 1945 ABC group *n* = 168, non-ABC group *n* = 1777				
ACEI	715 (36.8%)	113 (67.3%)	602 (33.9%)	<0.001
ARB	279 (14.3%)	55 (32.7%)	224 (12.6%)	<0.001
Aspirin	1058 (54.4%)	97 (57.7%)	961 (54.1%)	0.36
Beta-blocker	1133 (58.3%	119 (70.8%)	1114 (57.1%)	0.001
Verapamil or Diltiazem	164 (8.4%)	11 (6.5%)	153 (8.6%)	0.36
Other calcium channel blocker	160 (7.9%)	13 (7.7%)	147 (8.3%)	0.81
Clopidogrel	213 (11%)	22 (13.1%)	191 (10.7%)	0.35
Diuretics	949 (48.8%)	101 (60.01%)	848 (47.7%)	0.002
Digoxin	702 (36.1%)	55 (32.7%)	647 (36.4%)	0.34
Statin	938 (48.2%)	169 (97.0%)	775 (43.6%)	<0.001
Other lipid-lowering drug	29 (1.5%)	7 (4.2%)	22 (1.2%)	0.003
Warfarin	1049 (51.9%)	155 (92.3%)	894 (50.3%)	<0.001
Other anticoagulant	88 (4.5%)	13 (7.7%)	75 (4.2%)	0.036
Amiodarone	178 (9.2%)	27 (16.1%)	151(8.5%)	0.001
Flecainide	14 (0.7%)	1 (0, 6%)	13 (0.7%)	0.84
Propafenone	34 (1.7%)	1 (0, 6%)	33 (1.9%)	0.23
Sotalol	13 (0.7%)	2 (1.2%)	11 (0.6%)	0.38

Abbreviations: ACEI—angiotensin-converting-enzyme inhibitors, ARB—angiotensin receptor blockers, BMI—body mass index, LVEF—left ventricular ejection fraction, TIA—transient ischemic attack.

**Table 2:** ABC pathway compliance.

Study Groups	Compliance	Non-Compliance
A	1118 (55.3%)	903 (44.7%)
B	1518 (75.1%)	503 (24.9%)
C	388 (19.2%)	1575 (77.9%)
**ABC**	**168 (8.3%)**	**1853 (91.7)**

Abbreviations: A—avoid stroke, B—better symptoms management, C—cardiovascular and other comorbidities. The full ABC pathway compliance was highlighted in bold.

**Table 3:** Clinical outcomes at six-months and one-year follow-up.

Risk Factors	All-Cause Mortality				Composite Outcome			
	6 Months		1 Year		6 Months		1 Year	
	OR (95% CI)	*p* Value	OR (95% CI)	*p* Value	OR (95% CI)	*p* Value	OR (95% CI)	*p* Value
AF type (paroxysmal vs. persistent/permanent)	1.11 (0.92–1.33)	0.3	1.26 (1.07–1.48)	0.006	1.29 (1.13–1.46)	<0.001	1.33 (1.19–1.49)	<0.001
Renal dysfunction	3.04 (1.86–4.97)	<0.001	3.05 (1.91–4.89)	<0.001	1.94 (1.30–2.92)	0.001	1.8 (1.22–2.65)	0.003
Dyslipidemia	1.20 (0.83–1.73)	0.32	0.97 (0.71–1.33)	0.85	1.26 (0.99–1.62)	0.06	1.24 (0.99–1.54)	0.06
Use of aspirin	1.33 (0.93–1.90)	0.12	1.45 (1.07–1.97)	0.018	1.41 (1.11–1.79)	0.006	1.42 (1.14–1.76)	0.001
Major bleeding	1.84 (0.84–4.04)	0.13	1.74 (0.87–3.51)	0.12	2.13 (1.22–3.72)	0.008	3.09 (1.81–5.28)	<0.001
ABC Compliance	0.31 (0.13–0.77)	0.013	0.46 (0.25–0.86)	0.015	0.49 (0.31–0.79)	0.003	0.53 (0.36–0.80)	0.002

Abbreviations: CI—confidence interval, OR—odds ratio, TIA—transient ischemic attack.

**Table 4:** Relationship between ABC pathway components criteria and clinical outcomes.

Fulfilled Criteria	All-Cause Mortality		Composite Outcome	
	1 Year		1 Year	
	OR (95% CI)	*p* Value	OR (95% CI)	*p* Value
AB	0.78 (0.58–1.06)	0.12	0.75 (0.61–0.92)	0.006
AC	0.95 (0.62–1.46)	0.83	1.0 (0.74–1.36)	0.99
BC	0.73 (0.47–1.13)	0.16	0.68 (0.50–0.92)	0.013

Abbreviations: CI—confidence interval, OR—odds ratio.

## Supplementary Materials

The following are available online at https://www.mdpi.com/2077-0383/9/5/1286/s1, Figure S1. Classification of patients included in the study according to the risk for stroke based on the CHA_2_DS_2_-VASc score. Figure S2. A simplified methodology scheme of the evaluation of compliance of AF management with ABC pathway components. Table S1. Anticoagulation management. Table S2. Management of comorbidities according to the current guidelines.
