# Supplementary material for: Compliance of Atrial Fibrillation Treatment with the Atrial Fibrillation Better Care (ABC) Pathway Improves the Clinical Outcomes in the Middle East Population: A Report from the Gulf Survey of Atrial Fibrillation Events (SAFE) Registry
Source: J Clin Med. 2020 Apr 29;9(5):1286. doi: 10.3390/jcm9051286 (PMC7287832; doi:10.3390/jcm9051286)

# Supplementary materials

**Table s1. Anticoagulation management.**

| | **High stroke risk**<br>n= 1490 (73.7%) | **Low stroke risk**<br>n=531 (26.3%) |
|---|---|---|
| No treatment | 91 (6.4%) | 69 (13.1%) |
| Single antiplatelet therapy | 403 (28.3%) | 197 (37.6%) |
| Dual antiplatelet therapy | 97 (6.8%) | 18 (3.4%) |
| Anticoagulant only | 469 (33%) | 207 (39.3%) |
| Dual antithrombotic therapy | 294 (20.7%) | 28 (5.4%) |
| Triple antithrombotic therapy | 37 (2.6%) | 4 (0.8%) |
| Other combinations | 29 (2.2%) | 2 (0.4%) |

High stroke risk: ♀ $CHA_sDS_s$-VASc ≥2, ♂ $CHA_sDS_s$-VASc ≥1

Low stroke risk: ♀ $CHA_sDS_s$-VASc <2, ♂ $CHA_sDS_s$-VASc

**Table s2. Management of comorbidities according to the current guidelines.**

| | Hypertension (n = 1029) | HF (n = 524) | CAD (n= 552) | Diabetes mellitus (n=603) | Dyslipidaemia (n= 662) |
|---|---|---|---|---|---|
| **ACEI (%)** | 462 (44.9%) | 285 (54.4%) | 274 (49.6%) | | |
| **ARB (%)** | 232 (22.5%) | 95 (18.1%) | 129 (23.4%) | | |
| **Verapamil/diltiazem (%)** | 110 (10.7%) | | 42 (7.6%) | | |
| **Other Calcium Channel blocker (%)** | 144 (14%) | | 77 (13.9%) | | |
| **Beta-blocker (%)** | 624 (60.6%) | 263 (50.2%) | 332 (60.1%) | | |
| **Digoxin (%)** | | 331 (63,2%) | | | |
| **Diuretic (%)** | 552 (53.6%) | 478 (91.2%) | | | |
| **Aspirin (%)** | | | 372 (67.4%) | | |
| **Clopidogrel (%)** | | | 125 (22.6%) | | |
| **Statin (%)** | | | 440 (79.7%) | | 559 (84.4%) |
| **Other lipid-lowering agent (%)** | | | 12 (2.2%) | | 16 (2.4%) |
| **Diet (%)** | | | | 60 (10%) | |
| **Insulin therapy (%)** | | | | 162 (26.9%) | |
| **Oral antidiabetic drugs (%)** | | | | 409 (67.8%) | |

Abbreviations: ACEI-angiotensin-converting-enzyme inhibitors, ARB-angiotensin receptor blockers, CAD-coronary artery disease, HF-heart failure,

**Figure s1.**

**Classification of patients included in the study according to the risk for stroke based on the $CHA_2DS_2$-VASc score.**

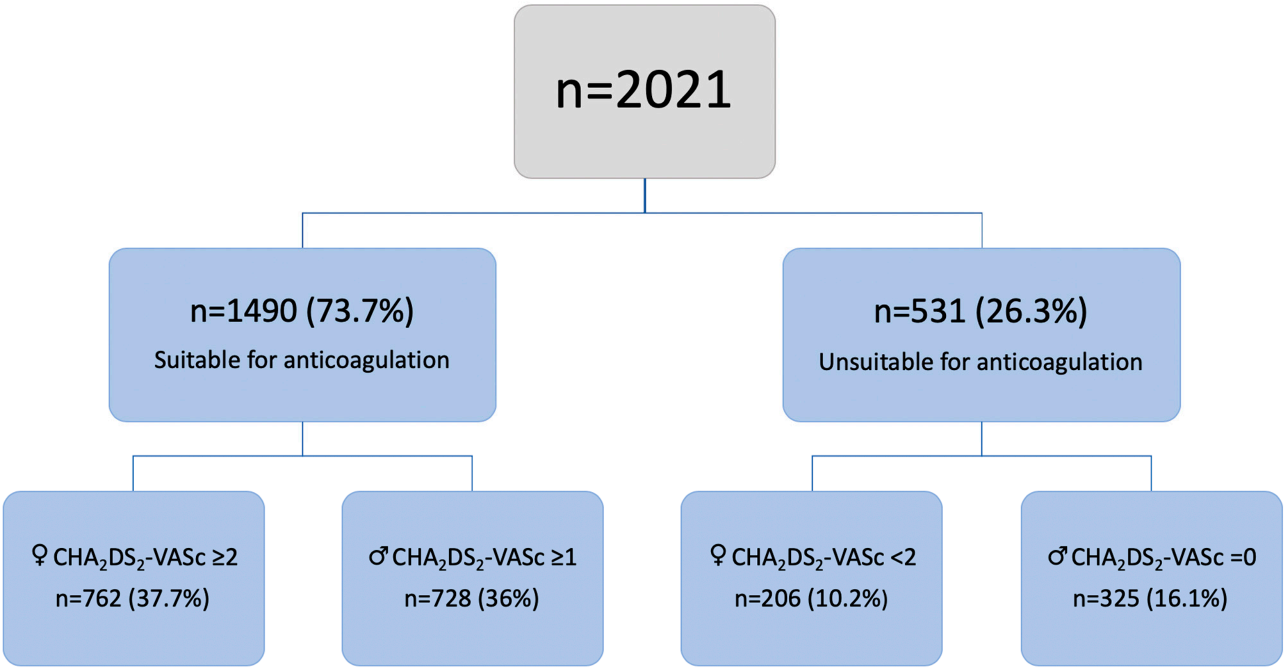

**Figure s2. A simplified methodology scheme of the evaluation of compliance of AF management with ABC pathway components.**

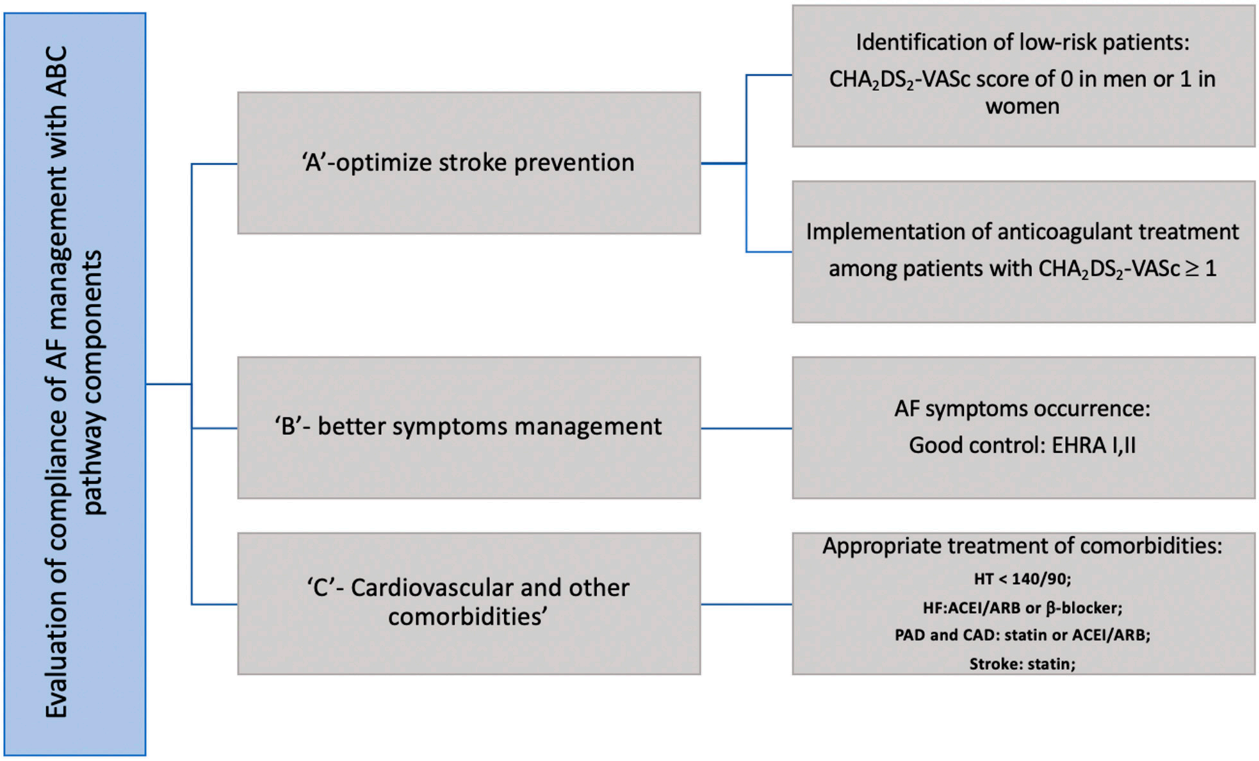

Supplement: Supplementary file 1 [file jcm-09-01286-s001.pdf]
